# The methylation site cg06972019 regulates the succinylation-related gene ENO1 to inhibit the occurrence of erectile dysfunction

**DOI:** 10.1186/s41065-025-00543-z

**Published:** 2025-08-26

**Authors:** Jian Wang, Siyuan Ye, Maoxiao Xu, Mengru Sun, Yang Lu, Zhenrong Piao, Fengmeng Teng, Maosen Zhang

**Affiliations:** 1 Affiliated Hospital of Nanjing University of Chinese Medicine, Nanjing, 210000 China; 2 The First Clinical Medical College of Nanjing, University of Chinese Medicine, Nanjing, 210000 China; 3 NanJing JiangNing Hospital of Chinese Medicine, Nanjing, 210000 China; 4 School of Clinical Medicine, Chengdu University of Traditional Chinese Medicine, Sichuan, 610000 China; 5 Changzhou Traditional Chinese Medicine Hospital, Changzhou, 213000 China

**Keywords:** Erectile dysfunction, Mendelian randomization, Cardiovascular diseases, ENO1

## Abstract

**Background:**

This study aims to explore the causal relationship between the expression of succinylation-related genes and erectile dysfunction (ED).

**Method:**

Through a literature review, we identified 19 succinylation-related genes and intersected them with cis-expression Quantitative Trait Loci (cis-eQTL) data from the eQTLGen Consortium, ultimately selecting 16 genes with available cis-eQTL data. Subsequently, we downloaded genomic data related to erectile dysfunction (ED) from 223,805 European male participants in the IEU OpenGWAS project and performed a two-sample Mendelian Randomization (MR) analysis. Summary-based Mendelian Randomization (SMR) analysis and ELISA testing further confirmed the statistical association between ENO1 gene expression and ED risk. Mediation analysis was used to explore the potential regulatory role of DNA methylation in the relationship between gene expression and ED.

**Result:**

Through MR analysis, a significant causal relationship between the ENO1 gene and ED was identified. The results indicated that the expression of the ENO1 gene has a significant causal effect on the risk of ED (OR: 1.2388, 95% CI: 1.0708–1.4332, *p* < 0.05). SMR analysis further confirmed the causal relationship between ENO1 gene expression and ED (SMR_p-value = 0.0040). Mediation analysis suggested that the methylation site cg06972019 may inhibit the occurrence of ED by regulating ENO1, with the mediation proportion accounting for 67.6% of the total effect (*P* = 0.0013). ELISA results showed that the serum ENO1 levels in ED patients were significantly higher than those in the healthy control group (*p* < 0.05), validating the potential role of ENO1 in ED.

**Conclusion:**

This study revealed the potential causal relationship of the ENO1 gene in the development of ED through Mendelian Randomization and SMR analysis, further validating the association between gene expression and ED. The overexpression of the ENO1 gene may be regulated by the methylation site cg06972019. These findings provide new insights into the molecular mechanisms of ED and may offer new biomarkers for the early diagnosis and targeted treatment of ED.

## Introduction

By 2025, the global prevalence of erectile dysfunction (ED) is projected to rise to 322 million individuals [1]. Traditionally more common in older populations, contemporary research demonstrates that ED incidence is increasingly affecting younger men, becoming a significant health concern for this demographic [2]. The etiology of ED is complex, encompassing neurogenic, vascular, psychogenic, and endocrine factors, among others [3]. Vascular factors are widely regarded as a major causative element, as the stability of cavernosal hemodynamics and the integrity of vascular endothelial function play crucial roles in the erectile process [4, 5].

Notably, ED and cardiovascular diseases (CVDs) share several common risk factors, including diabetes, hypertension, dyslipidemia, smoking, and advanced age [6]. Furthermore, endothelial dysfunction is recognized as a shared pathological basis for both conditions. Consequently, a growing body of research suggests a close association between ED and CVDs [7]. A study evaluating the prevalence of ED in patients presenting with acute chest pain and confirmed coronary artery disease by angiography utilized semi-structured interviews with 300 patients to collect medical and sexual health histories, combined with tools like the International Index of Erectile Function. The study found that nearly 70% of ED patients experienced erectile dysfunction before the onset of coronary heart disease symptoms, suggesting ED may serve as an early warning signal for coronary artery disease [8]. Another prospective Australian study, analyzing questionnaire data alongside hospitalization and mortality records, examined the relationship between ED severity and the risk of CVDs and all-cause mortality. The results indicated that more severe ED was associated with a higher risk of CVDs and death, further underscoring the strong link between ED and CVDs [9].

Acyl modification is a post-translational covalent modification process involving the addition of acyl groups (e.g., acetyl, succinyl, fatty acyl) to amino acid residues of proteins. This alters protein structure, stability, function, and interactions, thereby influencing diverse biological processes such as cellular metabolism, gene expression, and signal transduction [10, 11]. Succinylation, a specific type of acyl modification, involves the transfer of a succinyl group from succinyl-CoA to lysine residues on proteins [12]. Research indicates that succinylation, by regulating the activity of enzymes involved in fatty acid β-oxidation, can lead to reduced fatty acid oxidation capacity. This increases dependence on glycolysis, resulting in lactate accumulation and exacerbating cardiac injury during ischemia [13]. Additionally, succinylation enhances the activity of succinate dehydrogenase, promoting succinate accumulation and thereby intensifying oxidative stress and worsening cardiac damage. Studies have shown that deficiency of the mitochondrial desuccinylase Sirt5 leads to elevated cardiac succinylation levels, triggering myocardial metabolic disturbances and accelerating the progression of cardiovascular disease [14, 15]. Thus, succinylation plays a pivotal role in the initiation and progression of cardiovascular diseases. Given the bidirectional relationship between ED and CVDs, their shared risk factors and pathological mechanisms, and the significant role succinylation plays in regulating cardiovascular metabolism and function, its potential role in the pathogenesis of ED warrants further investigation.

In recent years, genomic research has increasingly focused on establishing causal relationships between genes and diseases, becoming a vital direction in biomedical research. Genetic methodologies like Mendelian randomization (MR) also offer novel perspectives for revealing potential causal links between specific gene expression and disease development [16]. Through literature review, this study collected information on succinylation-related genes. By integrating Mendelian randomization analysis and mediating effect analysis, it aims to investigate the potential mechanisms by which succinylation-related genes contribute to the occurrence and development of ED, providing a foundation for subsequent research.

## Materials and methods

### Data sources

**Fig. 1:**
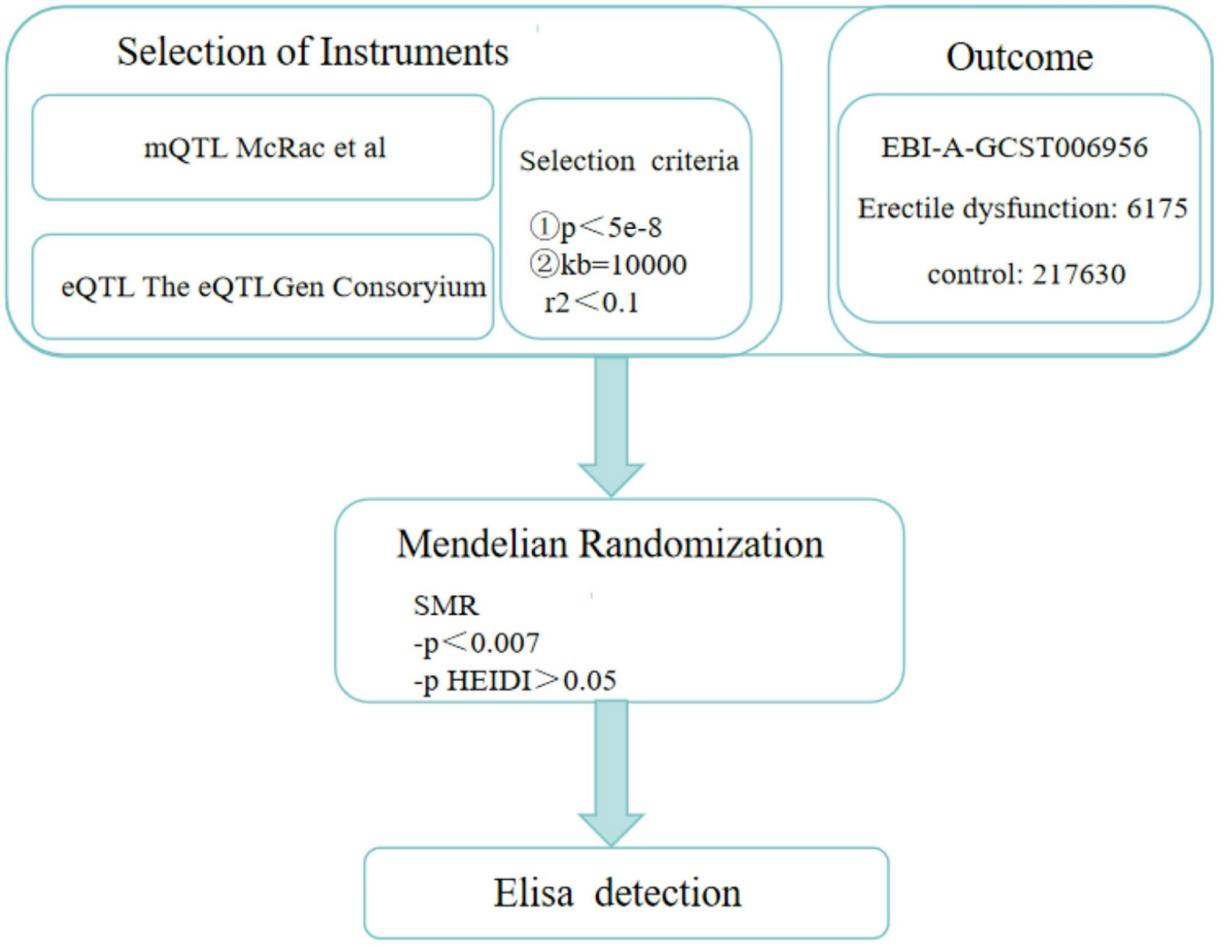
Flowchart

This study identified 19 succinylation-related genes through a literature review; the specific gene list is provided in Table 1 [17–21] (see Fig. 1 for the Flowchart). Expression quantitative trait loci (eQTL) represent genomic regions where genetic variation is associated with gene expression levels. eQTL are categorized as cis-eQTL or trans-eQTL [22]. The eQTLGen Consortium is an international collaborative project focused on identifying relationships between genotypes and gene expression levels through large-scale gene expression data analysis. The cis-eQTL data used in this study, downloaded from the eQTLGen database (https://www.eqtlgen.org/cis-eqtls.html), were exclusively derived from blood samples. Instrumental variables (SNPs) were selected using the following parameters: *p*-value < 5e-8, clumping window = 10,000 kb, and r² < 0.1. This yielded 15,695 cis-eQTL genes. These genes were intersected with the 19 succinylation-related genes, resulting in 16 succinylation genes possessing cis-eQTL data. Furthermore, Genome-Wide Association Study (GWAS) data for ED were obtained from the IEU OpenGWAS project database (https://gwas.mrcieu.ac.uk/), dataset ID “EBI-A-GCST006956”. This dataset originates from a study by Bovijn et al. that merged three population cohorts, encompassing 223,805 European male individuals (6,175 cases and 217,630 controls).

**Table 1:** Succinylation related genes

Succinylation related genes
ACOX1, CPT1A, CTBP1, ENO1, FBN1, GLS, H2AX, HADHA, HAT1, KAT2A, LDHA, OGA, PGAM1, PKM, S100A10, SDHA, SIRT5, SOD1, TAGLN2

### Two-sample MR analysis

We employed a two-sample MR design. Genetic variants selected as instrumental variables (IVs) must satisfy three core assumptions [23]: (1) Strong association with the exposure; (2) Independence from confounders; (3) Association with ED solely through the exposure. Cis-eQTL data corresponding to the succinylation genes served as the exposure variable, and ED GWAS summary statistics served as the outcome variable. Analyses were conducted using the TwoSampleMR package in R. The strength of the genetic instruments was assessed using the F-statistic; IVs with an F-statistic > 10 were retained to mitigate weak instrument bias [24]. To ensure analytical robustness, multiple statistical methods were applied: Inverse-Variance Weighted (IVW), Weighted Median (WM), MR-Egger, Weighted Mode, and Simple Mode. Given its high statistical power and robustness under valid instruments, IVW was designated as the primary analysis method [25]. A False Discovery Rate correction was applied to identify SNPs exhibiting significant causal associations with ED. Horizontal pleiotropy was evaluated using the MR-Egger intercept test. Heterogeneity among IVs was assessed using Cochran’s Q test. Sensitivity analysis was performed using the leave-one-out method to evaluate the influence of individual SNPs on the overall causal estimate and further assess result robustness. All statistical analyses were performed using R version 4.2.2.

### Summary-data-based mendelian randomization (SMR) analysis

To further investigate the causal relationship between exposure and outcome, we performed SMR analysis. SMR is a summary-statistics-based MR method that integrates GWAS and eQTL data to infer causal effects of gene expression levels on disease phenotypes [26]. Both SMR analysis and the heterogeneity in dependent instruments (HEIDI) test were conducted using the SMR software tool (version 1.3.1). A p_SMR value < 0.05 was considered statistically significant. Default parameter settings were used for the SMR analysis.

### Mediation analysis

A two-step MR approach was employed to explore whether ENO1 gene expression is mediated by DNA methylation. Methylation quantitative trait loci (mQTL) data were obtained from the GoDMC database (http://www.GoDMC.org.uk/). First, data for 19 methylation sites associated with the ENO1 gene were extracted. IVs were selected (*p*-value < 5e-8, clumping window = 10,000 kb, r² < 0.1), resulting in mQTL data for 9 sites used as the exposure, with ED data as the outcome. MR analysis using the TwoSampleMR package in R version 4.4.2 identified 7 mQTL sites significantly associated with ED. The total effect of the mQTL on ED was denoted as beta_all. The effect of the mQTL on ENO1 expression was denoted as beta1, and the effect of ENO1 expression on ED was denoted as beta2. The mediated effect was calculated as beta12 = beta1 * beta2. The mediation proportion was calculated as beta12_p = (beta12 / beta_all) * 100%, along with its corresponding *p*-value.

### Clinical sample collection

This study enrolled 49 male participants: 22 controls and 27 ED patients. Baseline information for all participants was collected by attending physicians during clinical consultations. Participants completed the International Index of Erectile Function-5 (IIEF-5) questionnaire; assessments were subsequently performed by the attending physicians. Participants were stratified based on IIEF-5 scores: ED group (IIEF-5 < 22) and control group (IIEF-5 ≥ 22). Exclusion criteria included: severe cardiovascular or cerebrovascular disease, metabolic syndrome, psychiatric disorders, alcoholism, genital trauma or deformity, recent ED treatment of any form, and recent use of medications known to inhibit erectile function. The study protocol was approved by the Clinical Research Ethics Committee of Jiangsu Provincial Hospital of Traditional Chinese Medicine (Approval No. 2024NL-309-01). The study strictly adhered to the principles outlined in the Declaration of Helsinki (1964).

### Serum ENO1 measurement by enzyme-linked immunosorbent assay (ELISA)

Fasting venous blood samples (3 ml) were collected from both patients and controls during their initial visit using coagulation-promoting tubes. Samples were allowed to clot at room temperature for 30 min, followed by centrifugation at 3500 rpm for 5 min. Serum levels of ENO1 were quantified using a commercially available ELISA kit (Jiangsu Meimian Industrial Co., Ltd., China). The assay procedure was strictly performed according to the manufacturer’s instructions.

## Results

### Identification of succinylation-related genes

Intersection analysis between succinylation-related genes and cis-eQTL gene data extracted from the eQTLGen database yielded 16 relevant genes.

### Two-sample MR and SMR analyses

In the two-sample MR analysis, using cis-eQTL data corresponding to succinylation as the exposure variable and ED data as the outcome variable, results indicated that ENO1 promotes ED development. The MR-Egger intercept test confirmed no significant pleiotropy (intercept *P* > 0.05), and Cochran’s Q test indicated no heterogeneity (*P* > 0.05). Sensitivity analysis using the leave-one-out method further supported the robustness of the results. SMR analysis, which detects causal relationships between gene expression and disease risk, revealed a statistically significant association between ENO1 and ED. ENO1 overexpression promotes ED occurrence (SMR_P = 0.0040, HEIDI_P = 0.2206, OR: 1.2388, 95% CI: 1.0708–1.4332).

### Mediation MR analysis

**Fig. 2:**
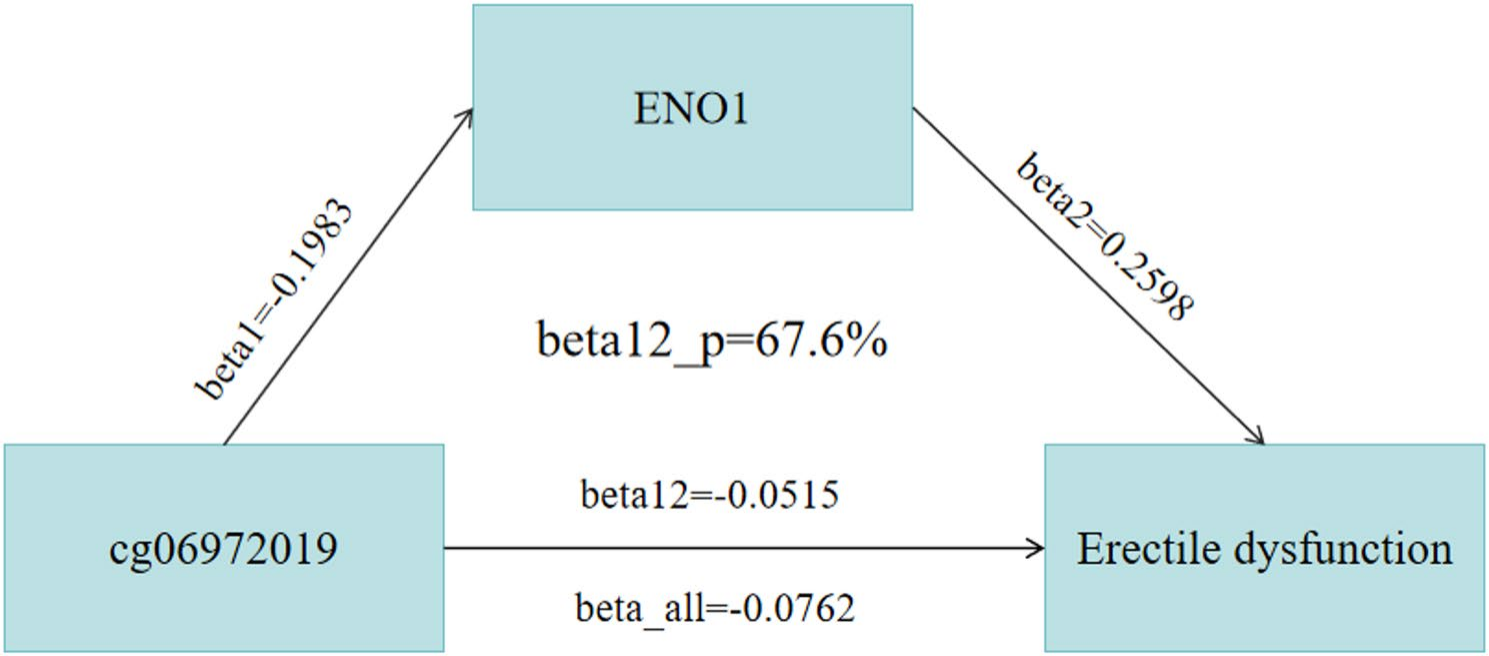
Mediation analysis model of DNA methylation, palmitoylation gene expression, and erectile dysfunction (ED) risk. beta 1: the effect of DNA methylation sites on ENO1 expression. beta 2: the effect of ENO1 expression on ED risk. beta 12: the total effect of DNA methylation on ED risk. Beta_all: The overall effect of DNA methylation on the risk of ED. beta12_p: mediation proportion

A two-step mediation MR analysis was conducted. First, 19 methylation sites related to the ENO1 gene were extracted. Tool variables were selected by setting parameters to *p*-value < 5e-8, clumping window: 10,000 kb, r2 < 0.1, resulting in 9 mQTL sites as the exposure variable. ED data was selected as the outcome. MR analysis was performed using the TwoSampleMR package in R version 4.4.2, yielding 7 mQTL data points associated with ED. After applying a corrected *P*-value (*P* < 0.007), one methylation site, cg06972019, showed statistical significance (*P* = 0.0022). In the mediation MR analysis, beta1 was − 0.1983, beta2 was 0.2598, the mediation effect beta12 was − 0.0515, the direct effect beta_all was − 0.0762, the mediation proportion beta12_p was 67.6%, and P was 0.0013 (see Figs. 2).

### Sensitivity analysis results

**Fig. 3:**
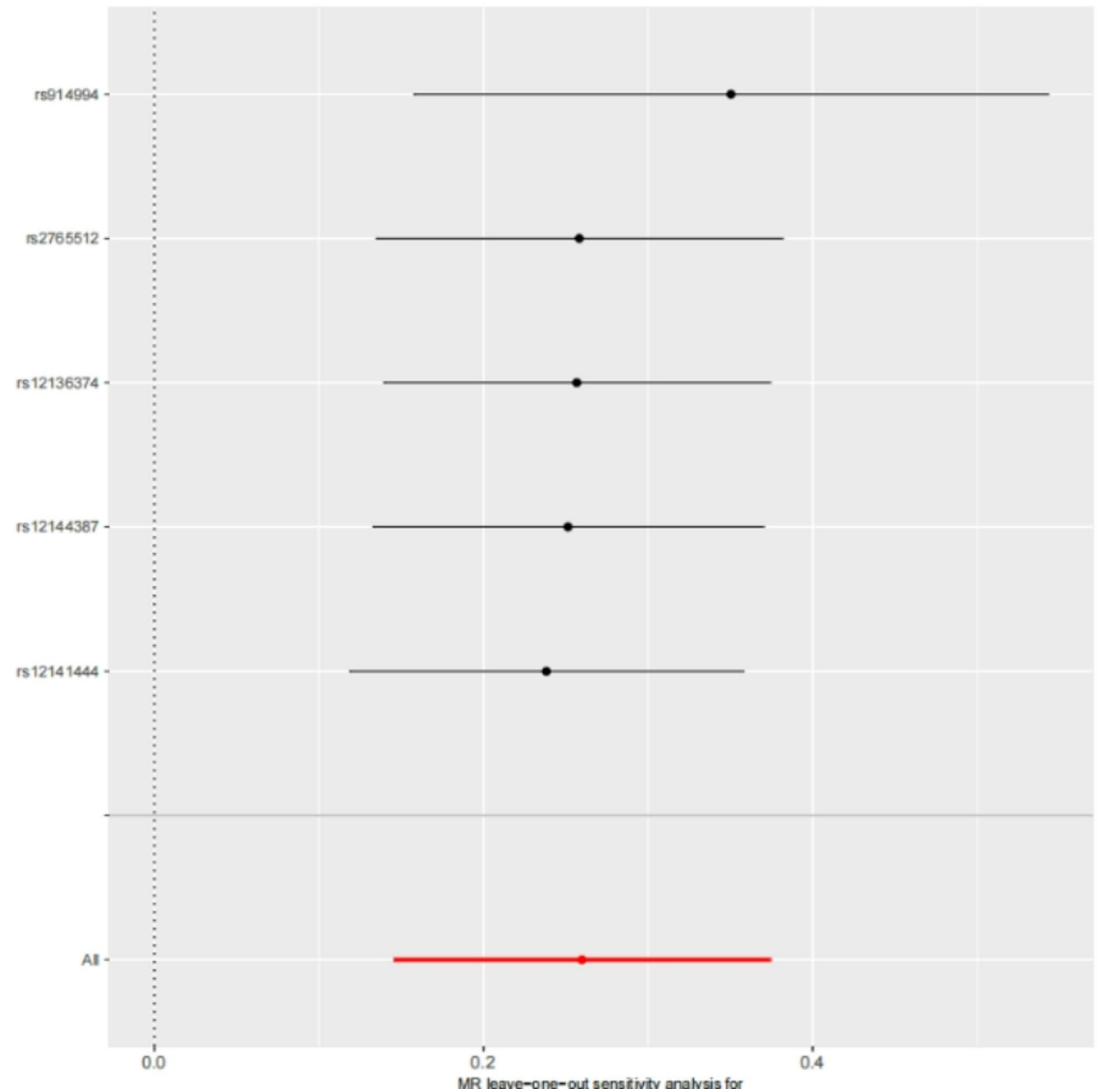
Leave-one-out diagrams for causal effects of ENO1 on Erectile dysfunction

**Fig. 4:**
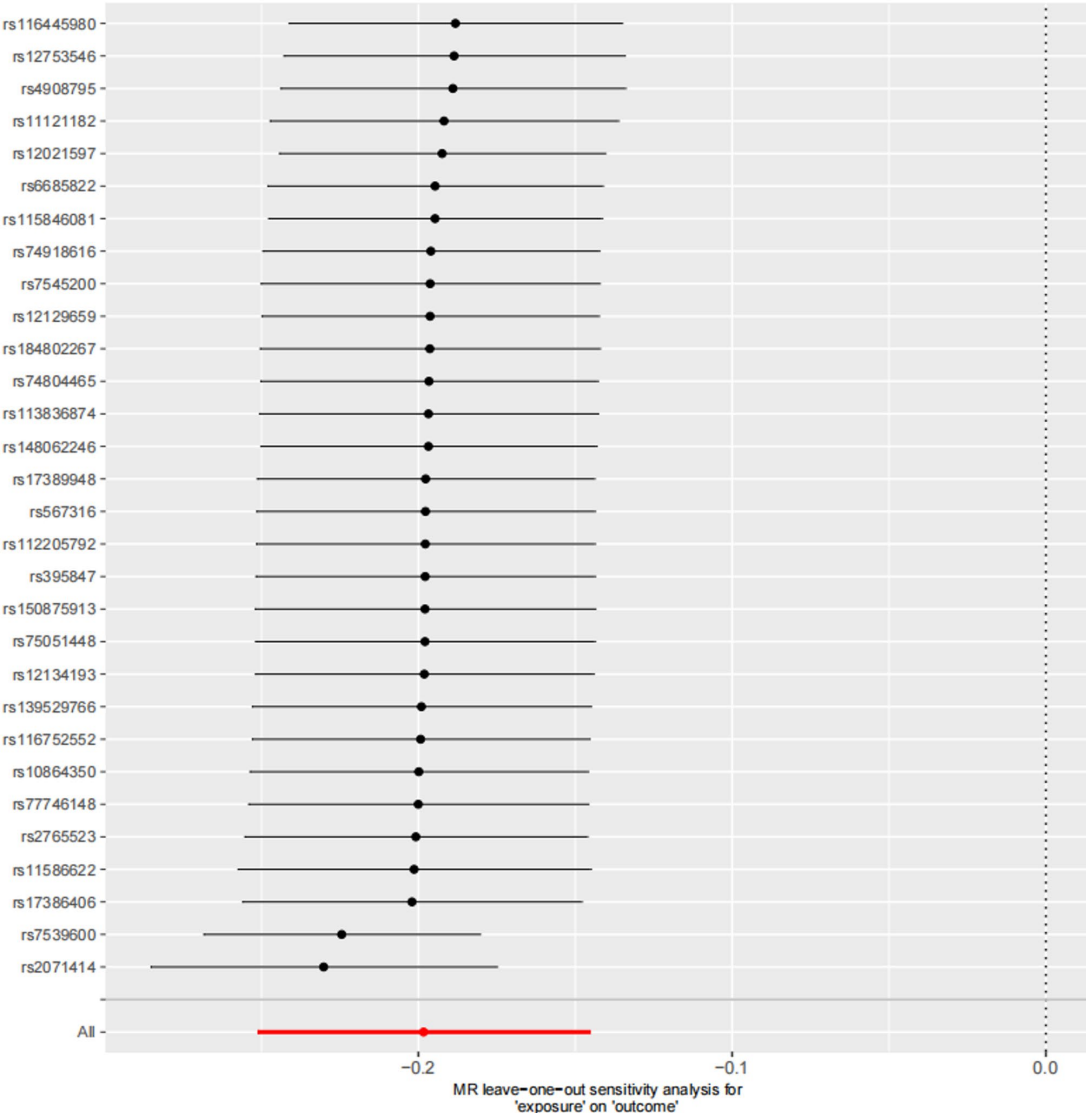
Leave-one-out diagrams for causal effects of cg06972019 on Erectile dysfunction

Sensitivity analyses were conducted to ensure the reliability and robustness of the MR results. The MR-Egger intercept test showed no statistically significant intercept (*P* > 0.05;see Tables 2 and 3), indicating no evidence of horizontal pleiotropy and confirming the validity of the MR core assumptions. Cochran’s Q test assessed heterogeneity among IVs, yielding non-significant results (*P* > 0.05;see Tables 2 and 3), thereby excluding potential bias from heterogeneity in causal estimates. Leave-one-out analysis was performed by iteratively removing individual SNPs and re-estimating the MR effect. Results demonstrated no substantial alteration in the direction or significance of the causal effect estimates upon exclusion of any single SNP (see Figs. 3 and 4), indicating that the primary conclusions were not driven by individual influential IVs.

**Table 2:** Mendelian randomization results show genes significantly associated with ED

Gene	OR (95%CI)	p_value (IVW)	MR-Egger intercept	Cochran’s Q
CPT1A	0.9632 (0.8786, 1.0559)	0.4238	0.0835	0.2797
CTBP1	1.0529 (0.9839, 1.1267)	0.1361	0.2829	0.8388
ENO1	1.2967 (1.1566, 1.4538)	8.47E-06	0.4456	0.7183
FBN1	0.8675 (0.7488, 1.0048)	0.0580	0.8377	0.4515
GLS	0.9459 (0.8061, 1.1099)	0.4954	0.3044	0.2581
HAT1	0.9072 (0.7580, 1.0858)	0.2882	0.6410	0.6637
PGAM1	1.0056 (0.9213, 1.0977)	0.9003	0.6636	0.4173
S100A10	1.1886 (0.8940, 1.5804)	0.2345	0.9530	0.8604
SIRT5	1.0156 (0.8299, 1.2428)	0.8807	0.3632	0.6698
TAGLN2	1.0353 (0.9151, 1.1712)	0.5815	0.2005	0.7657

**Table 3:** Mendelian randomization results show cg06972019 associated with ED

DNA methylation sites	OR (95%CI)	p_value (IVW)	MR-Egger intercept	Cochran’s Q
cg06972019	0.8201 (0.7778, 0.8646)	<0.0001	0.3438	0.1853

### ENO1 detection results

**Fig. 5:**
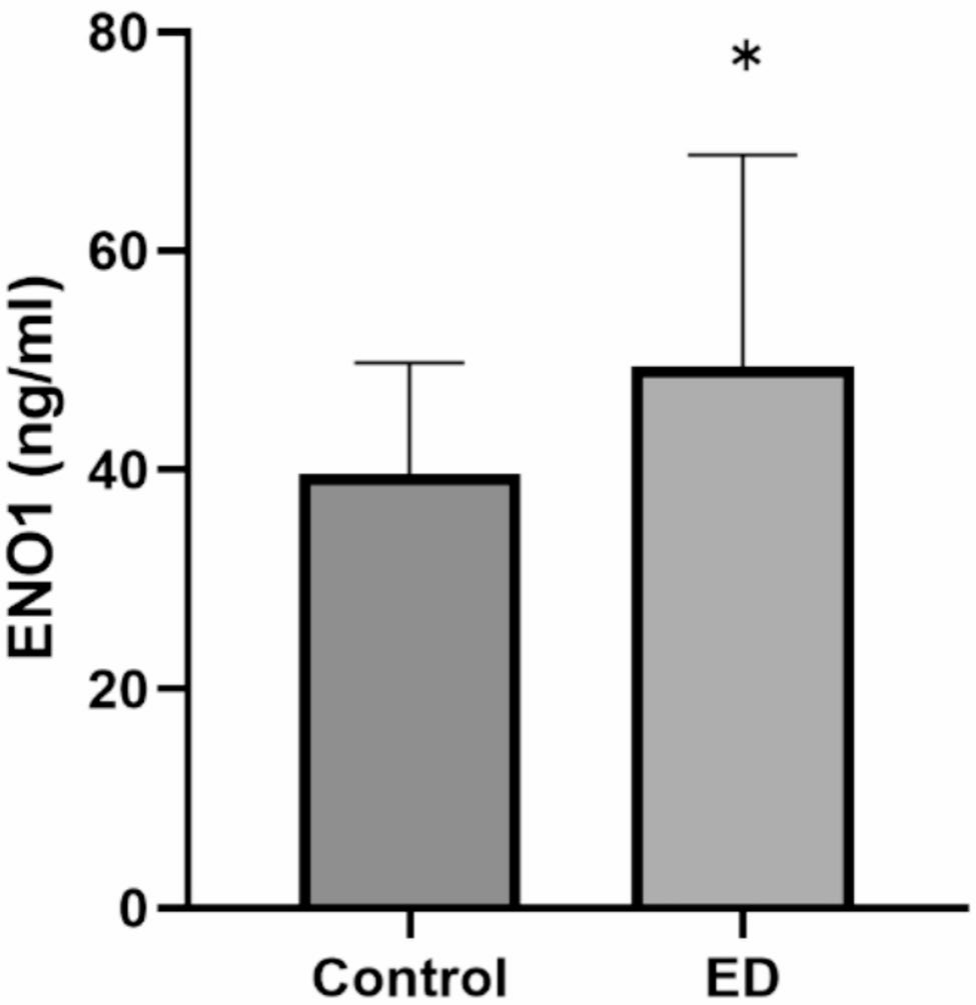
The comparison of ENO1 expression between the ED group and the control group

The results showed that the serum ENO1 levels in ED patients were significantly higher compared to the control group, and the difference was statistically significant (see Fig. 5).

## Discussion

This study aims to investigate the potential role of succinylation-related genes in ED risk, providing a foundation for subsequent experimental research. Succinylation, a significant form of post-translational modification, plays a critical role in CVDs by regulating processes such as energy metabolism, oxidative stress, and cellular signaling [12–15]. Given the well-established link between ED and cardiovascular health, and considering that CVDs can increase ED risk through various mechanisms affecting vascular endothelial function, hemodynamics, the nervous system, and hormonal levels, exploring the role of succinylation-related genes in ED offers a novel perspective for understanding ED pathogenesis. Furthermore, it provides preliminary data support for future investigations into the relationship between ENO1 succinylation and ED.

In this study, we first retrieved and compiled succinylation-related genes from PubMed and performed an intersection analysis with cis-eQTL data from the eQTLGen database. Subsequently, by integrating eQTL and GWAS data and employing MR and SMR analyses, we identified a significant association between the succinylation-related gene ENO1 and an increased risk of ED. To validate this association, we measured serum ENO1 levels in ED patients and controls, revealing significantly elevated ENO1 levels in the ED patient cohort. Further mediation analysis demonstrated that the methylation site cg06972019 reduces ED risk by suppressing ENO1 gene expression. These findings provide an initial theoretical basis for elucidating the role of ENO1 succinylation in ED pathogenesis and offer crucial clues for validating the potential function of ENO1 in ED.

ENO1 belongs to the widely expressed enolase family. Its encoded protein, α-enolase, is a core glycolytic enzyme that catalyzes the dehydration of 2-phosphoglycerate to phosphoenolpyruvate [27]. Beyond this fundamental metabolic function in the cytosol, cell surface ENO1 acts as a plasminogen receptor, mediating plasminogen activation and participating in extracellular matrix degradation, tissue remodeling, cell migration, and inflammatory responses. Notably, cytosolic ENO1 has also been implicated in maintaining mitochondrial membrane stability [28–30]. ENO1 is frequently overexpressed in various cancers, driving tumor progression through mechanisms including promotion of glycolysis (Warburg effect), enhanced proliferation, inhibition of apoptosis, facilitation of invasion and metastasis, and adaptation to hypoxic microenvironments [31–33]. Consequently, it is regarded as an important diagnostic biomarker and therapeutic target in oncology [28]. Additionally, aberrant ENO1 expression or function is closely associated with autoimmune diseases [34], cardiovascular diseases [35, 36], and neurological disorders [37].

Currently, direct evidence implicating ENO1 in the pathogenesis of erectile dysfunction is lacking. However, existing studies provide significant clues: ENO1 overexpression promotes excessive proliferation, angiogenesis, and aberrant adhesion in pulmonary arterial endothelial cells, disrupting endothelial homeostasis. More importantly, ENO1 can induce mitochondrial dysfunction by targeting and regulating mitochondria-related genes, triggering oxidative stress and cellular damage, thereby exacerbating endothelial dysfunction [38, 39]. This is highly relevant to the core pathological mechanism of ED. ED fundamentally arises from impaired relaxation of corpus cavernosum smooth muscle, a process critically dependent on a functionally intact NO-cGMP signaling pathway. Mitochondrial dysfunction is a key factor leading to the failure of this pathway [40]. Under the influence of risk factors such as inflammation, diabetes, and hypertension, excessive mitochondrial fission leads to network fragmentation, initiating a cascade of detrimental effects: ①Disruption of Energy and Calcium Homeostasis: Fragmented mitochondria exhibit drastically reduced ATP synthesis, compromising the energy supply required for smooth muscle relaxation. Concurrently, the loss of calcium buffering capacity disrupts the regulation of myocyte contraction/relaxation. ②Oxidative Stress Surge: Dysfunctional mitochondria generate excessive reactive oxygen species (ROS). Elevated ROS not only directly damage cellular components but also consume and inactivate the critical signaling molecule nitric oxide (NO). ③endothelial nitric oxide synthase (eNOS) Dysfunction: Mitochondrial damage and increased ROS cause uncoupling of eNOS, transforming it from an NO producer into a source of ROS, further diminishing NO bioavailability. These combined effects culminate in the failure of the NO-cGMP signaling pathway, ultimately hindering corpus cavernosum smooth muscle relaxation and blood perfusion. Furthermore, the role of glycolysis and related genes/miRNAs in diabetic ED has been documented [41]. Given the central role of ENO1 in glycolysis and its significant regulatory impact on mitochondrial function, its dysregulated expression may promote ED progression by mediating energy metabolism imbalance, exacerbating oxidative stress, and impairing endothelial cell function. Thus, ENO1 emerges as a key molecular target in ED pathogenesis. Therefore, in-depth investigation into the specific molecular mechanisms of ENO1 succinylation in ED is crucial for understanding ED pathophysiology and identifying potential therapeutic interventions. Based on these findings, our subsequent research will employ integrated cellular and animal models to focus on elucidating the role of ENO1 succinylation in the development and progression of ED.

This study employed a multi-omics MR approach, integrating genetic data from both mQTL and eQTL levels. This strategy enhanced statistical power and effectively controlled for confounding factors and reverse causality [42]. The MR analysis results demonstrated that overexpression of the ENO1 gene may increase the risk of developing ED. This finding was further validated by the SMR analysis. Mediation analysis revealed that the methylation site cg06972019 suppresses ENO1 expression, thereby reducing the risk of ED onset. This discovery provides a novel perspective on the potential association between the ENO1 gene and ED and lays a theoretical foundation for ED prevention and treatment. Future research could further validate the regulatory role of methylation site cg06972019 on ENO1 expression and elucidate its mechanistic role in ED using cellular experiments and animal models, with particular attention to succinylation modifications of ENO1 under diverse physiological and pathological states.

This study has several limitations. First, our findings rely on existing public databases, which may be subject to selection bias or data incompleteness, potentially affecting the generalizability and accuracy of the results. Although these databases encompass large-scale data, their predominant derivation from European populations limits extrapolation to other ethnic groups. Second, despite employing two-sample MR with multiple statistical approaches, potential biases from instrumental variable selection or pleiotropy could influence causal inferences. Future studies should refine IV selection strategies and analytical frameworks. Third, although mediation analysis incorporated DNA methylation’s role in regulating ENO1 expression, the methylation-gene expression relationship is compounded by environmental factors, cell-type heterogeneity, and genetic backgrounds. These complexities challenge the model’s ability to fully capture underlying biological mechanisms. Fourth, the limited clinical cohort size (*n* = 49) constrains the statistical power of the ELISA validation. We will expand sample sizes in future studies and perform post-hoc power analysis to confirm adequacy and optimize experimental design. Fifth, the association between ENO1 and ED lacks direct prior evidence; thus, the mechanistic links require experimental exploration. Finally, heterogeneity in sample sizes across integrated databases may impact statistical robustness. Validation in larger, multi-ethnic cohorts remains essential. These follow-up efforts will provide stronger experimental support for elucidating the role of ENO1 succinylation in ED pathogenesis.

## Conclusion

This study reveals the involvement of the ENO1 gene in ED pathogenesis. Overexpression of ENO1 may increase ED risk, while the methylation site cg06972019 exerts a protective effect by suppressing ENO1 expression. Furthermore, succinylation modifications likely play a pivotal regulatory role in ENO1 functionality, warranting in-depth exploration. These findings provide novel molecular insights into ED pathology and lay the groundwork for developing targeted prevention and therapeutic strategies.

## Data Availability

No datasets were generated or analysed during the current study.

## Abbreviations

ED: Erectile Dysfunction
CVDs: Cardiovascular diseases
MR: Mendelian randomization
eQTL: Expression Quantitative Trait Loci
SNPs: Instrumental variables
IVs: Instrumental variables
IVW: Inverse Variance Weighting
WM: Weighted Median
SMR: Summary-based Mendelian Randomization
HEIDI: Heterogeneity in dependent instruments
mQTL: Metabolite Quantitative Trait Loci
IIEF-5: International Index of Erectile Function-5
ROS: Reactive oxygen species
NO: Nitric oxide
eNOS: Endothelial nitric oxide synthase
GWAS: Genome-Wide Association Study
